# Supplementary material for: Angiotensin II Exaggerates SARS-CoV-2 Specific T-Cell Response in Convalescent Individuals following COVID-19
Source: Int J Mol Sci. 2022 Aug 4;23(15):8669. doi: 10.3390/ijms23158669 (PMC9368904; doi:10.3390/ijms23158669)
Supplement: Supplementary file 1 [file ijms-23-08669-s001.zip › ijms-1834261-supplementary.pdf]

Supplementary Figures

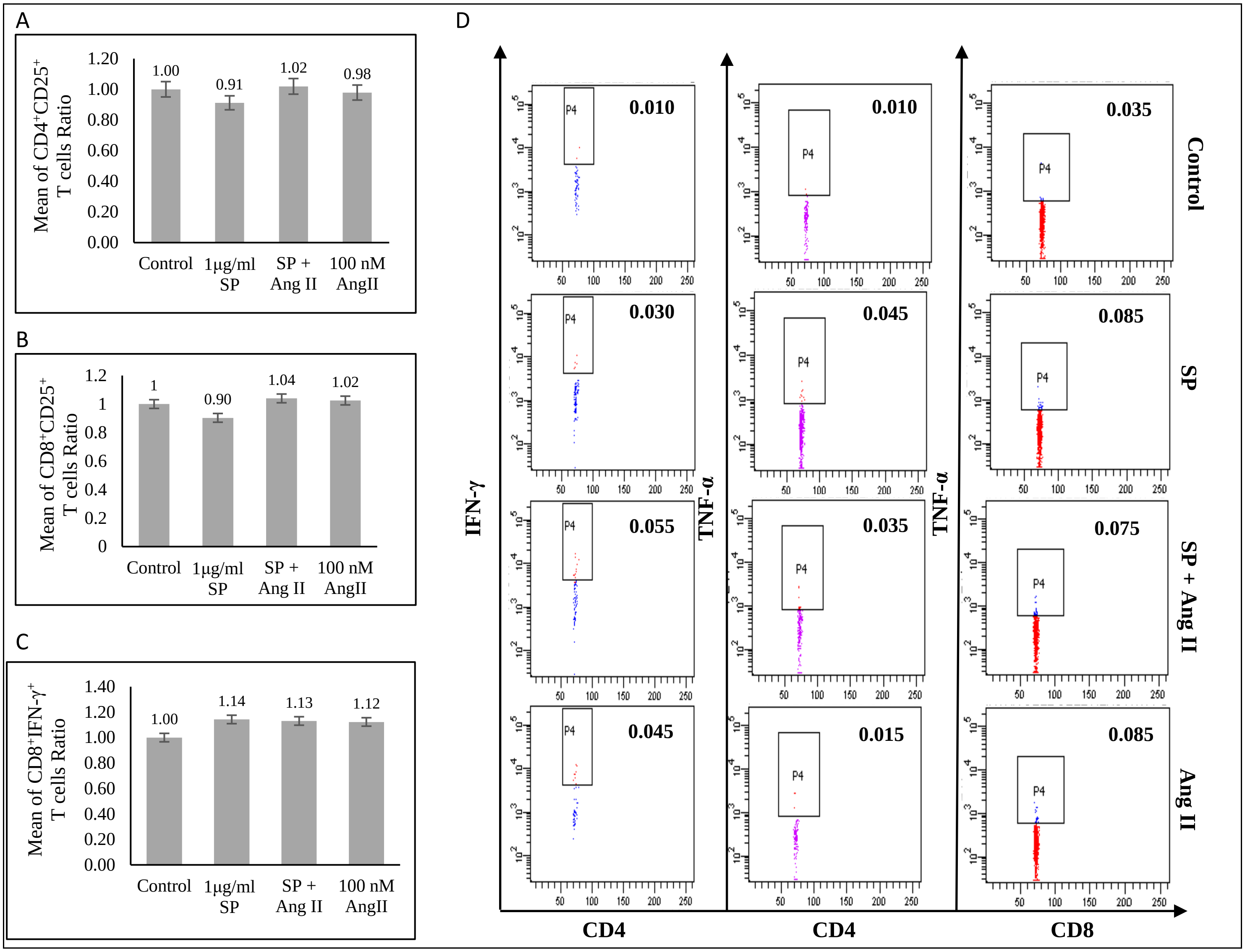

Figure S1: Activated T-cells markers of recovered COVID-19 patients (n = 5)
The ratio of $CD4^+$ and $CD8^+$ T-cells responses were measured as the mean of percentage of the cell surface marker positive T-cells in each group to the percentage of the cell surface marker positive T-cells in the control group after stimulation of PBMCs with SARS-COV-2 peptide pool (SP), Angiotensin II (Ang II) or both of SP and Ang II. **(A)** Cell surface $CD4^+CD25^+$ T-cells. **(B)** Cell surface $CD8^+CD25^+$ T-cells. **(C)** Ratio of $CD8^+$ T-cells producing intracellular IFN-γ.**(D)** FACS representative plot of intracellular cytokine production.

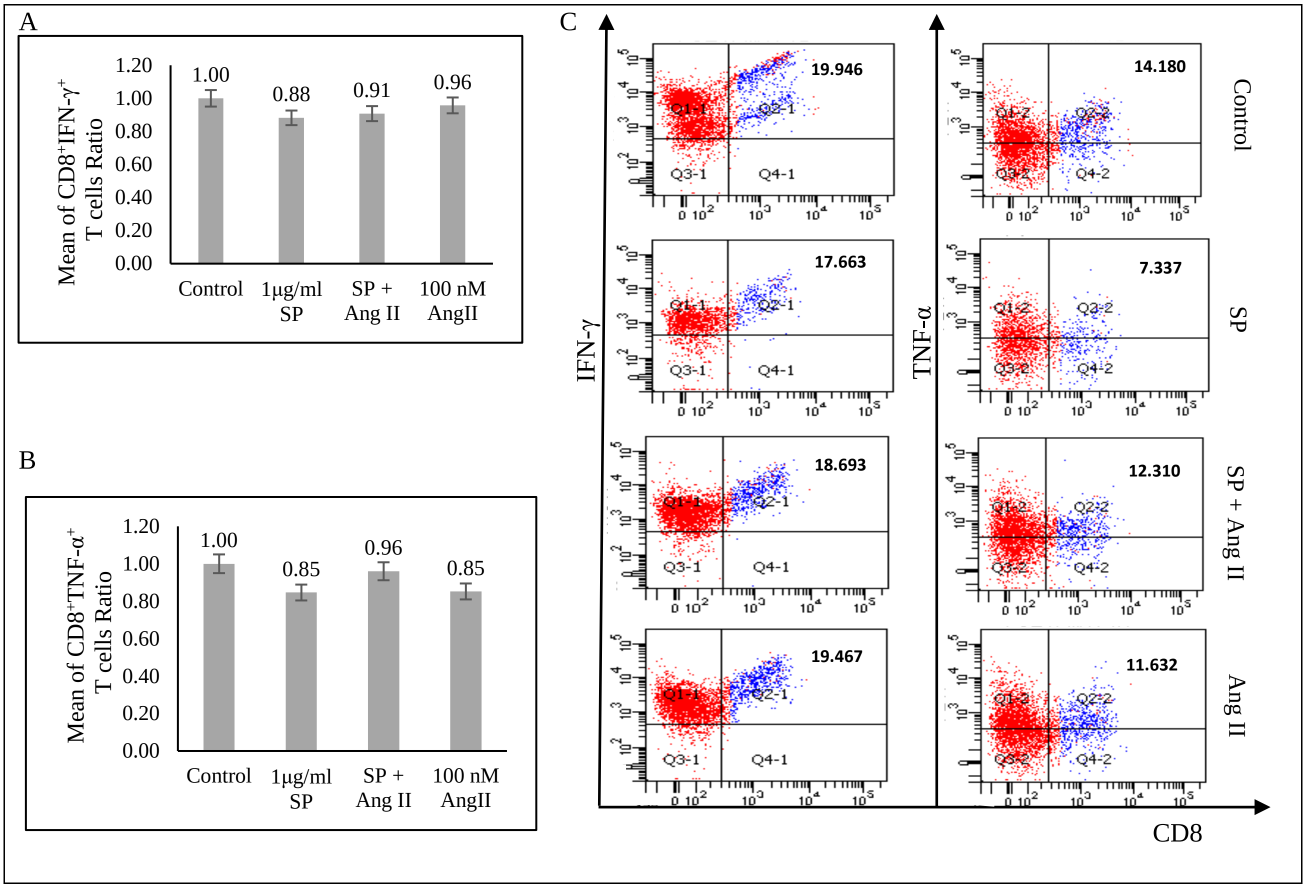

Figure S2: Activated T-cells markers of recovered COVID-19 patients (n = 5)

The mean of T-cells ratio were measured as the percentage of T-cells producing cytokine in each group to the percentage of T-cells producing cytokine in the control group after stimulation of PBMCs with SARS-COV-2 peptide pool (SP), Angiotensin II (Ang II) or both of SP and Ang II. Mitogenic stimulated $CD8^+$ T-cells producing **(A)** IFN-γ, **(B)** TNF-α. **(C)** FACS representative plot.

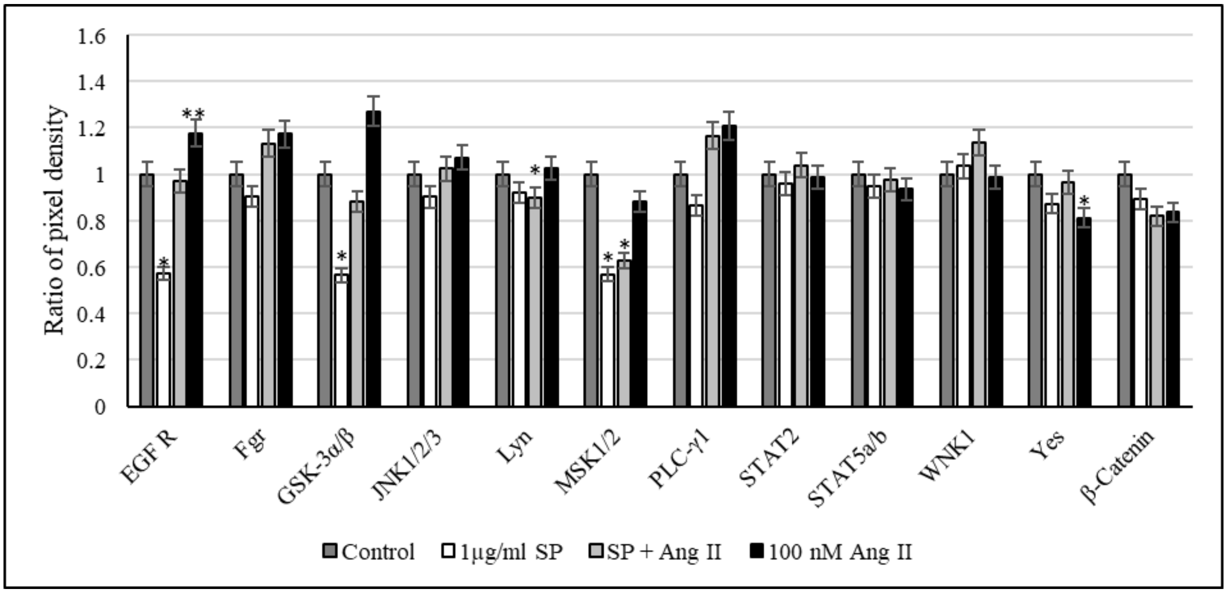

Figure S3. Phospho-kinase array analysis of PBMCs from recovered COVID-19 patients (n = 13) after stimulation of PBMCs with SARS-COV-2 peptide pool (SP), Angiotensin II (Ang II) or both of SP and Ang II. The ratio of pixel density was measured as the mean of pixel density of each group to the mean of pixel density of the control group. * denotes P < 0.05 vs. control group. ** denotes P < 0.05 vs. 1μg/ml SP group.

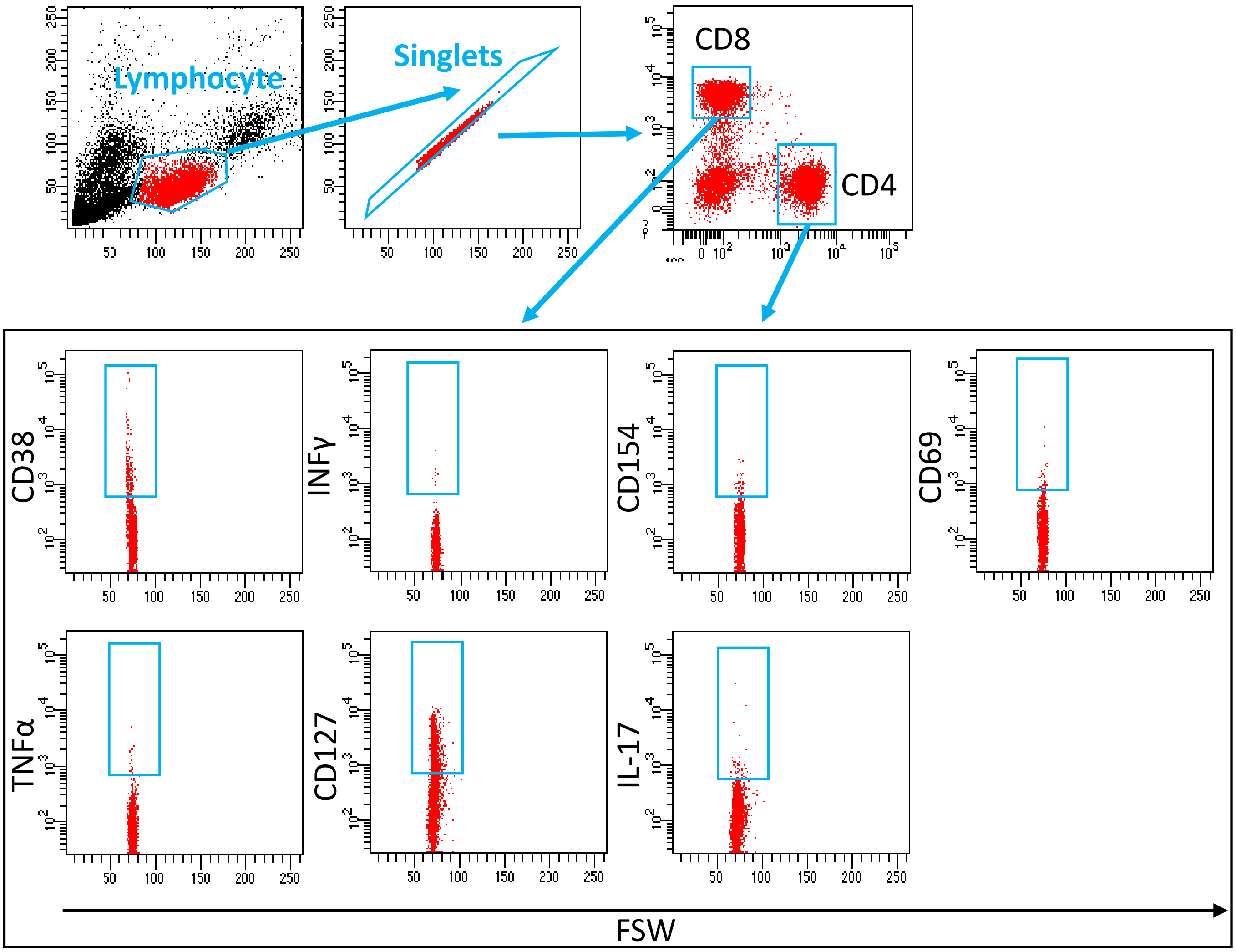

Figure S4: Flowcytometry gating strategy of T-cells from recovered COVID-19 patients.
